# Novel role of autophagy-associated Pik3c3 gene in gonadal white adipose tissue browning in aged C57/Bl6 male mice

**DOI:** 10.18632/aging.101426

**Published:** 2018-04-25

**Authors:** Amiya Kumar Ghosh, Theresa Mau, Martin O’Brien, Raymond Yung

**Affiliations:** 1 Division of Geriatric and Palliative Medicine, Department of Internal Medicine, University of Michigan, Ann Arbor, MI 48109, USA; 2 Geriatric Research, Education and Clinical Care Center (GRECC), VA Ann Arbor Health System, Ann Arbor, MI 48105, USA

**Keywords:** aging, adipose tissue, inflammation, ER-Stress, autophagy

## Abstract

Adipose tissue dysfunction is associated with inflammation, metabolic syndrome and other diseases in aging. Recent work has demonstrated that compromised autophagy activity in aging adipose tissue promotes ER stress responses, contributing to adipose tissue and systemic inflammation in aging. Phosphatidylinositol 3-kinase catalytic subunit type 3 (Pik3c3) is an 887 amino acid lipid kinase that regulates intracellular membrane trafficking and autophagy activity. To address the mechanistic link between autophagy and ER stress response in aging adipose tissue, we generated a line of adipose tissue-specific *Pik3c3* knock out (~mutant mice) with the *Fabp4* (Fatty acid binding protein 4) promoter driven *Cre* recombinase system. We found elevated ER stress response signaling with reduced autophagy activity without any significant change on adiposity or glucose tolerance in early life of Pik3c3 mutant mice. Interestingly, middle- and old-aged mutant mice exhibited improved glucose tolerance (GTT) and reduced adiposity compared to age and sex-matched littermates. In addition, adipose tissue-specific *Pik3c3* mutants display reduced expression of adiposity-associated genes with the signature of adipose tissue browning phenotypes in old age. Overall, the results suggest that altered adipose tissue characteristics due to autophagy inhibition early in life has beneficial effects that promote adipose tissue browning and improves glucose tolerance in late-life.

## Introduction

Chronic low-grade inflammation of white adipose tissue (WAT) is considered as one of the root causes of insulin resistance in aging, along with other age-associated diseases. Recent work from our group indicated that compromised autophagy is linked to elevated ER stress responses that contributed to the inflammation of WAT in aging [1]. Our previous works have examined the expressions of ER stress response genes in young and old animals in basal state or under the influence of chemical inducer/inhibitor of ER stress response [2]. Results of differential expressions of autophagy genes in mice of varying ages suggested a plausible link between compromised WAT autophagy activity and inflammation in aging [1].

Macro-autophagy (hereafter autophagy) plays an essential role in cellular homeostasis and stress adaptation, deficiency of which promotes multiple chronic diseases in aging [3]. Autophagy is a complex and dynamic membrane trafficking process that executes the delivery of intracellular content to lysosomes for degradation. A fully executed autophagy process includes the formation of double membraned autophagosomes, the fusion of autophagosomes to late endosomes/lysosomes, and the digestion of the enclosed content by lysosomal hydrolases [4]. Autophagy is constantly maintained at the basal level, and is up-regulated in response to stress conditions such as nutrient and energy limitation, hypoxia, and DNA damage. Cellular and tissue homeostasis is maintained with autophagy process by eliminating damaged organelles and misfolded proteins and dysregulation of which is implicated in developmental defects and multiple age-associated diseases [3,5-9].

Pik3c3, the mammalian orthologue of yeast Vps34 (vesicular protein sorting 34), is a Class III phosphoinositide 3-kinase (PI3K) that phosphorylates phosphatidylinositol to generate phosphatidylinositol 3-phosphate [PI(3)P], a phospholipid central to membrane trafficking and nucleation in autophagy. The localized production of PI(3)P acts to recruit effector proteins containing FYVE or PX domains that control membrane docking and fusion during the formation of internal vesicles [10]. Vps34 is the only PI3K identified in yeast thus far, and its essential role in vacuolar protein delivery was initially described through yeast genetics studies [11]. In metazoans, Vps34 has been found to play a critical role in endocytosis, autophagy, and TOR activation [12,13]. The essential role of Vps34 in autophagy has been examined largely through the use of the pharmacological inhibitors; wortmannin and 3-methyladenine (3-MA). However, because of the lack of specificities of these inhibitors, the precise role of Vps34 in autophagy remains unclear. It was only recently that the physiological role of mammalian Vps34/Pik3c3 has been investigated in mouse gene knockout studies [14]. Surprisingly, it was reported that although genetic deletion of Vps34 in sensory neurons leads to disruption of the endocytic pathway, the autophagy pathway is still intact in these cells, raising the question as to whether Vps34 is necessary for autophagy in mammals [14]. On the other hand, genetic deletion of the *Pik3c3* gene in the heart and liver also suggested an essential role of Vps34/Pik3c3 in controlling vesicular trafficking and autophagy in tissue-specific homeostasis and functions [15].

The role of autophagy in WAT inflammation has been examined in an ATG7-knock out mouse model, with beneficial effects on adipose tissue differentiation program in obesity [16]. On the other hand, an alternative pathway which is ATG5/ATG7 independent has also been identified, regulated by the Unc-51-like kinase 1 (Ulk1) and beclin1 [17] or by ubiquitin-like activating enzyme (Uba1) [18]. These reports strongly suggest redundancy in mammalian autophagy pathways. Therefore, gene disruption of autophagy initiation of the Vps34/Pik3c3 pathway in the context of adipose tissue biology will likely shed light on the importance of autophagy pathways in aging adipose tissue inflammation. In this study, we have generated adipose tissue-specific Pik3c3 mutant mice with the use of *Fabp4-*Cre recombinase system, as Fabp4 is exclusively expressed in adipocytes and in the stromal cell progenitors of white adipose tissue [19] Effects of Pik3c3 mutation is characterized in young animals followed by analysis of adiposity and glucose tolerance in middle-and old-aged mice.

## RESULTS

### Generation and characterization of adipose tissue-specific Pik3c3-mutant mice

**Figure 1:**
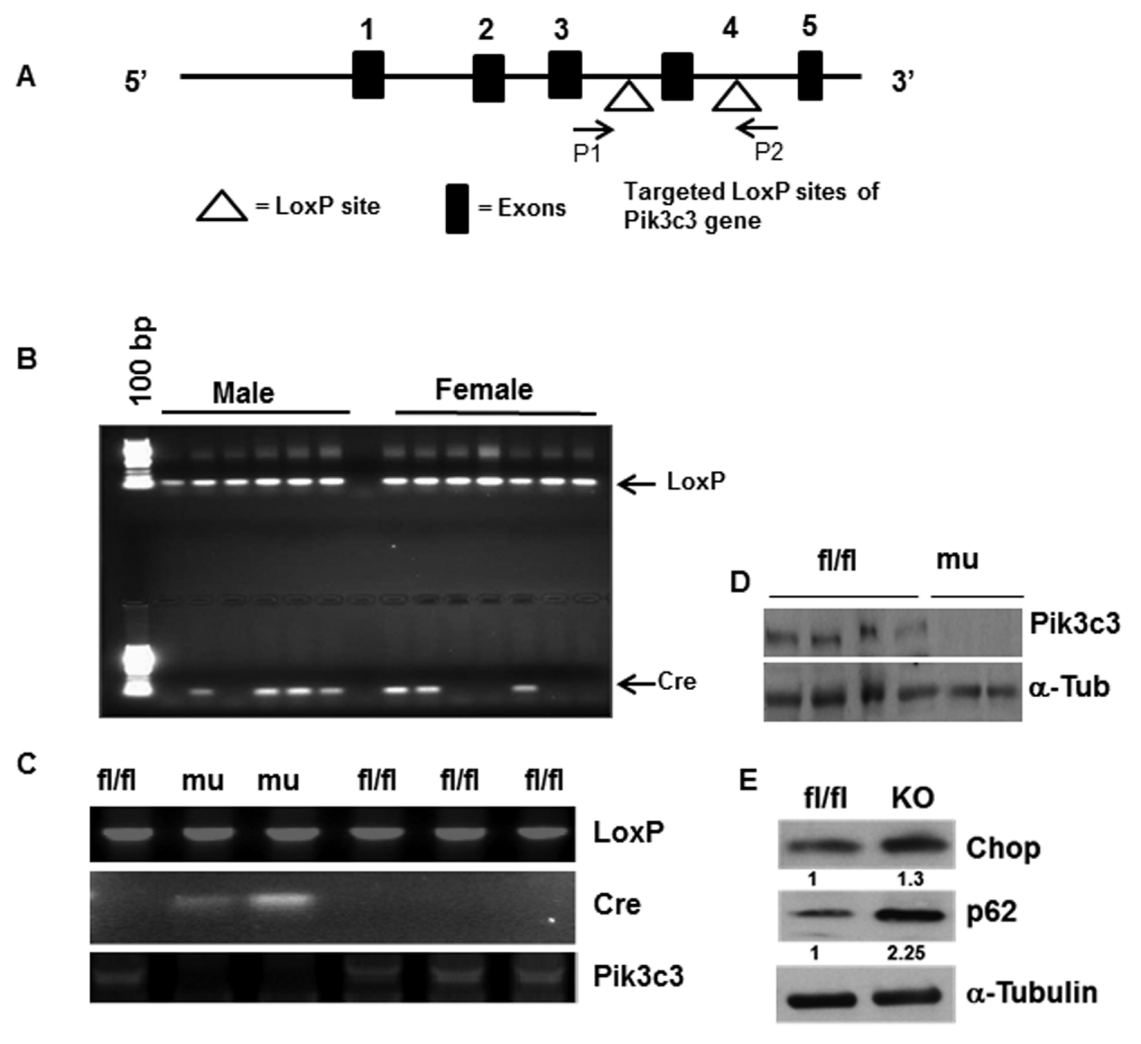
**Generation and characterization of adipose tissue specific Pik3c3-mutant mice.** Adipose tissue specific *Pik3c3* mutant mice were generated by breeding homozygous floxed *vps34/Pik3c3* (*Pik3c ^^fl/fl^^*) mice in which exon 4 of *Pik3c3* gene is flanked by *LoxP* sites (**A**) with fatty acid-binding protein 4-Cre recombinase (*Fabp4-Cre*) mice. (**B**) Genotype analysis was performed by using PCR primers for LoxP and Cre-recombinase and chromosomal DNA of tail biopsies were used as template. (**C**) LoxP and Cre expressions were analyzed using chromosomal DNA from adipocytes, and by using LoxP and Cre primers in PCR reactions. Deletion of exon 4 of *Pik3c3* gene was also confirmed by Pikc3c specific primers (third panel). (**D**) Western blot analysis of PIK3c3 in the adipocyte lysates from the fl/fl and Pik3c3 mutant mice. (**E**) Representative data of western blots of Chop and p62 on the adipocyte lysates from fl/fl and mutant mice (n=6). Relative band intensities were determined by densitometry measurements and normalized with corresponding α-tubulin band intensities and presented at the bottom of each band.

Pik3c3 mutant mice were generated by breeding Pik3c3-floxed female mice (where exon 4 of the *Pik3c3* gene flanked by *LoxP* sites) with male *Fabp4-Cre* (Fig. 1A). When exposed to adipose tissue-specific Cre recombinase (Fabp4-Cre), exon 4 of *Pik3c3* is excised, causing a frame-shift mutation leading to deletion of 755 of 887 amino acids of *Pik3c3* gene product. Mice were genotyped using chromosomal DNA from tail biopsies. As expected, all the littermates were LoxP positive but about 50% of them are positive for Cre recombinase gene both in males and females (Fig. 1B). Similar genetic analyses of LoxP and Cre expressions were conducted using chromosomal DNA from adipocytes fraction (Fig. 1C), confirming the gene mutation at the tissue level. Adipose tissue- specific mutation of Pik3c3 was also validated by the absence of Pik3c3 band for the mice expressing Cre recombinase using exon4 specific primers for *PiK3c3* gene (Fig. 1C).

We confirmed Pik3c3 deletion in the mutants by analyzing protein expression in adipose tissue lysates (Fig. 1D). As per our expectation, Pik3c3 protein band was absent in the mutants. We then examined the effect of the *Pik3c3* mutation on ER stress response and autophagy activity in the VAT of young male mice. Protein analysis of adipose tissue lysates showed elevated ER stress protein Chop expression and increased accumulation of autophagy substrate p62 protein (Fig. 1E), indicating that the adipose tissue-specific mutation of *Pik3c3* gene leads to autophagy inhibition with elevated ER stress response.

### Adipose tissue specific mutation of Pik3c3 alters adiposity and glucose tolerance in middle and old-aged mice

**Figure 2:**
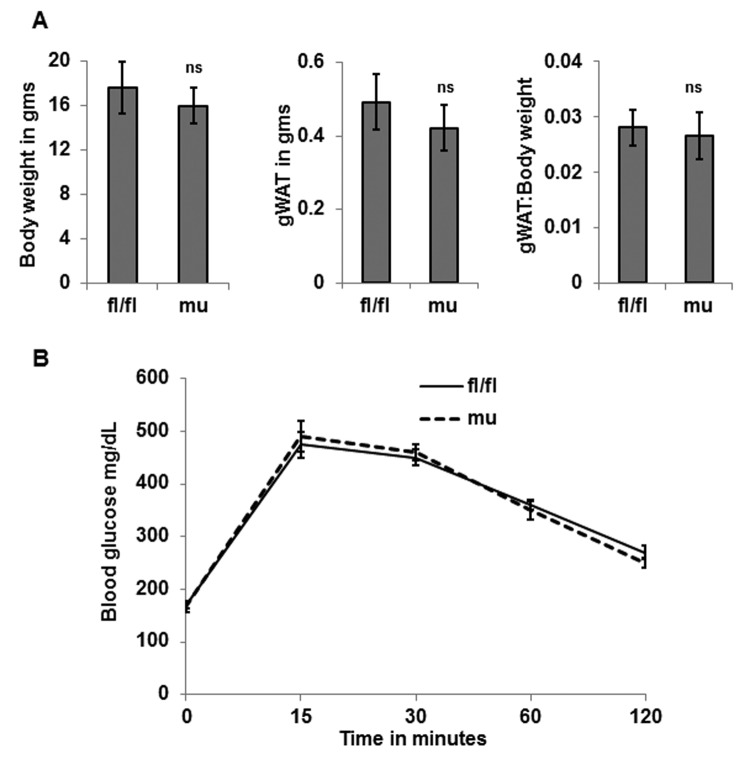
**Pik3c3 mutation has no effects on adiposity or glucose tolerance in young age.** Total body weight and gWAT and gWAT: body weight ratio of young (4 MO) of fl/fl and mutant mice (n=5 for each group) were plotted (**A**). Blood glucose levels were obtained from GTT and plotted (**B**). The significance levels were analyzed by unpaired Student’s t-test using means and SDs where * p<0.05 was considered as significant and ns=not significant.

**Figure 3:**
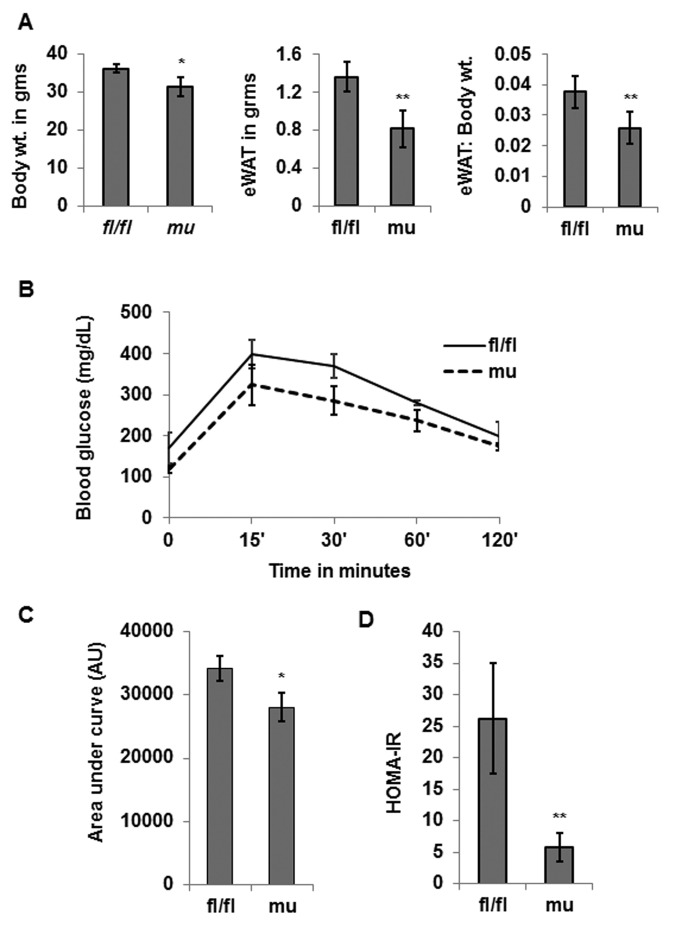
**Pik3c3 mutant middle aged mice exhibit reduced adiposity and improved glucose tolerance.** Total body weight, gWAT and gWAT: body weight ratio of middle aged (12 MO) fl/fl or mutant mice (n=5 for each group) were plotted (**A**). Blood glucose levels in GTT were plotted in (**B**) and area under the curve was plotted in (**C**). The significance levels were analyzed by unpaired Student’s t-test using means and SDs and designated as *p<0.05, **p<0.01, or ***p<0.001 and ns=not significant (p>0.05).

**Figure 4:**
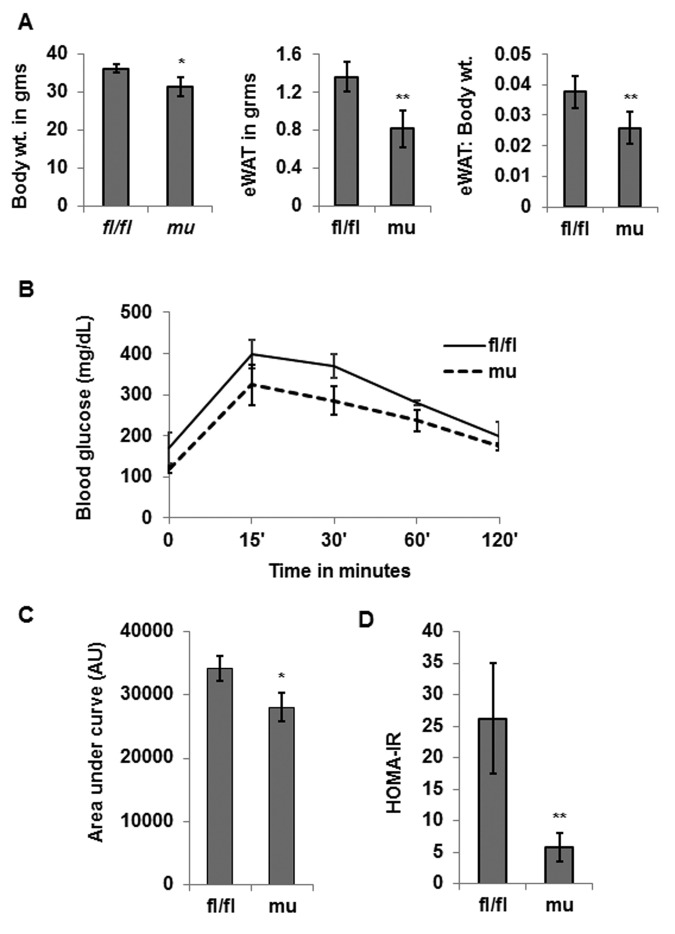
**Decreased adiposity and increased glucose tolerance in aging Pik3c3 mutant mice.** Total body weight and gWAT and gWAT: body weight ratio of male (24 MO) fl/fl and mutant mice (n=4 for each group) were plotted (**A**). Blood glucose levels in GTT were plotted (**B**) and area under the curve was plotted in (**C**). Insulin resistance was calculated by homeostatic model assessment of (HOMA) using fasting glucose and fasting insulin levels and plotted in D. The significance levels were analyzed by unpaired Student’s t-test using means and SDs and designated as *p<0.05, **p<0.01, or ***p<0.001 and ns=not significant (p>0.05).

To determine whether Pik3c3 mutation has any deleterious effects on adiposity or glucose metabolism, we measured the body weight, and performed glucose tolerance test (GTT) and weighed epididymal fat fads of young (4 mo.) male mice. We found no significant differences in the body weight, fat mass or in glucose tolerance between the fl/fl control and Pik3c3 mutant mice (Fig. 2). However, when analyzed the middle-aged (12 mo.) mice, we noticed significant differences in body weight, fat mass and glucose tolerance between fl/fl and Pik3c3 mutants (Fig. 3). Surprisingly, Pik3c3 mutant mice were leaner and performed better in GTT. We next analyzed body weight, gonadal fat mass and glucose tolerance in 24 month old male mice. Consistent with data from middle-aged mice, we found decreased body weight and fat mass with increased glucose tolerance in Pik3c3 mutants mice compared to their floxed littermates (Fig.4). Additionally, Pik3c3 mutants were less resistant to insulin as homeostasis model of insulin resistance (HOMA-IR) were significantly reduced (Fig. 4D). To reconcile our unanticipated observations, we hypo-thesized that: Pik3c3 mutation inhibited adipogenesis during normal aging process.

### Pik3c3 mutation leads to reduced adipogenesis in old mice

**Figure 5:**
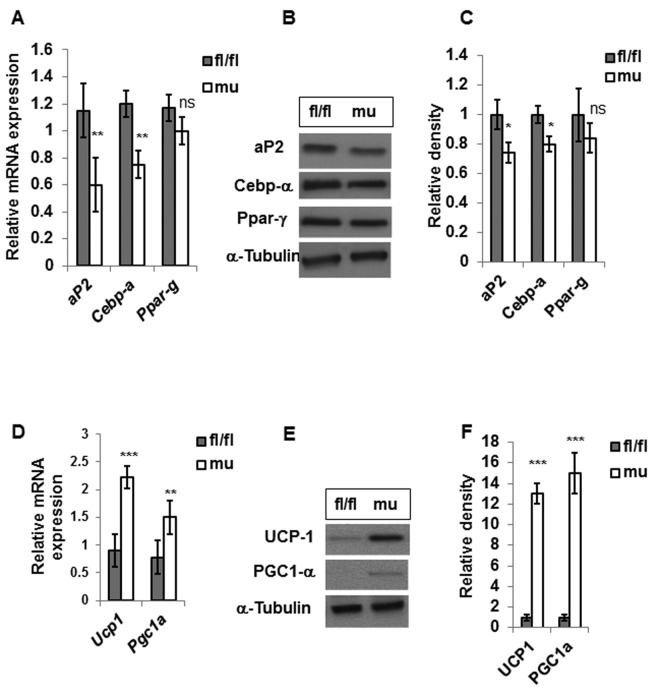
**PIK3c3 mutation reduced adipogenesis and enhanced browning in the gWAT of old mice** Expression of adipogenic factors (*aP2, Cebp-α* and *Ppar-γ*) were evaluated in 24 MO fl/fl and Pik3c3 mutant male mice (n=4 for each group). Both mRNA (**A**) and protein expression (**B**) were plotted. Relative intensity of protein bands were normalized with the corresponding α-tubulin bands and plotted (**C**). Expressions of *Ucp1* and *Pgc1a* mRNA (**D**) protein expressions were presented in (**E**). Relative intensities of protein expression were presented in (**F**). The significance levels were analyzed by unpaired Student’s t-test using means and SDs and designated as *p<0.05, **p<0.01, or ***p<0.001 and ns=not significant (p>0.05).

We analyzed the expressions of adipogenic genes (*aP2, Cebp-a, Ppar-γ*) in the gWAT of old-aged male mice. Analysis of mRNA showed diminished expressions of *aP2* and *Cebp-a* but not Ppar-g in gWAT of mutant mice compared to fl/fl littermates (Fig.5A). Furthermore, protein analyses confirmed reduced protein expressions of aP2 and Cebp-α but no significant change of Ppar-γ in Pik3c3 mutant mice (Fig. 5B & 5C).

### Pik3c3 mutant mice exhibit characteristics of brown fat in gWAT

To corroborate decreased adiposity and improved GTT in Pik3c3 mutant mice at old age, we hypothesized that Pik3c3 mutation enhanced brown adipose tissue development that improved glucose metabolism. We analyzed the expressions of *Ucp1* and *Pgc1a* genes that are signature markers for brown adipose tissue. Our results demonstrated elevated mRNA expressions of *Ucp1 and* Pgc1a in the gWAT of Pik3c3 mutant mice (Fig.5 D). Protein analyses of gWAT lysates showed elevated abundance of UCP1 and PGC1-α in Pik3c3 mutants compared to the fl/fl mice (Fig. 5E & 5F).

## DISCUSSION

Adipose tissue is at the crossroad of longevity and age-associated diseases involving inflammation and metabolic dysfunction. Longevity is extended with interventions that limit visceral fat development, such as: a) caloric restriction [20]. b) fat cell insulin receptor knock out (FIRKO), insulin receptor substrate (IRS-1) or S6 kinase 1 knockout mice models [21,22]. c) growth hormone receptor knock out (GHRKO) mice model [23]. d) rapamycin treatment [24,25], and by e) surgical removal of visceral fat [26,27]. To understand the molecular basis of adipose tissue inflammation, our work on gWAT across the lifespan have provided strong evidence of autophagy impairment [1] as a basis of elevated ER stress response which in turn promotes gWAT inflammation in aging mice. Recently, we have demonstrated that proficient autophagy activity and reduced senescence in gWAT were associated with improved glucose tolerance in aging *Tlr4*-knockout mice [28]. Much of the previous conclusions were derived by comparing expressions of ER stress response genes in young or old animals in basal state or under the influence of chemical inducers for ER stress response [29]. Similarly, differential expressions of autophagy genes in mice of varying ages suggested a causal association of autophagy activity with WAT inflammation in aging [1].

In this study we generated an adipose tissue-specific Pik3c3/Vps34 deficient mutant mouse strain (Fig. 1). Interestingly, we found significant differences in adiposity or glucose tolerance in middle and old-aged mice, but no difference in the young age (Fig. 3 & Fig. 4 vs Fig. 2). Our characterization of young mice suggested that the Pik3c3 mutation displayed elevated CHOP and p62 accumulation in the gWAT (Fig. 1E), but without affecting overall adiposity or glucose tolerance (Fig. 2). However, we observed improved glucose tolerance and reduced adiposity in the middle aged Pik3c3 mutant mice that indicated improved metabolic profile of these mice. More importantly, this improved GTT and reduced adiposity persisted till the old age in the Pik3c3 mutants.

We next examined if blocking autophagy may lead to decreased adipogenesis. Our analyses on gWAT of aging mice revealed that adipogenic factors (aP2, Cebp-α and Ppar-γ) are indeed reduced in the mutant mice compared to their control littermates (Fig 5A). These results correlated well with the observed adiposity data. Furthermore, expression data of brown adipose tissue markers (Ucp1 & Pgc-1α) indicated increased development of brown adipose tissue in the aging Pik3c3 mutant mice. Interestingly, similar observation has been described in adipocyte-specific autophagy related 7 knock out (ATG7 KO) model in the context of diet-induced obesity study [16]. Therefore, our observations are consistent with the published observations with the exception that we did not find any differences in adiposity or GTT in the young mice.

A possible mechanism of reduced adiposity in the aging Pik3c3-mutants could be explained by block of autophagy leading to the elevation of ER stress with elevated CHOP expression. Elevated CHOP expression might predispose adipocytes precursors to reduced adipogenesis, which is in agreement with the reports demonstrating increased CHOP expression/ER stress block adipogenesis by forming heterodimers with C/EBP (CCAAT/enhancer-binding protein) family members [30,31]. Inhibition of adipogenesis was reflected in the gWAT weight and in body weight measurements of the middle aged and the old aged mice. This was further supported by the reduced expression profile of known adipogenic genes (aP2, Cebp-α and Ppar-γ) in the old Pik3c3 mutants. We observed a significant decrease in the levels of aP2 and Cebp-α in mutants, but not Ppar-γ, compared to their control littermates. Taken together, overall expression of adipogenic factors were significantly reduced in the Pik3c3 mutant mice.

Recent studies have demonstrated that aP2 acts as an adipokine to regulate systemic metabolism. aP2-deficient mice have improved adipose and liver function and increased insulin sensitivity in the context of high fat diet (HFD) and in genetic mouse models [32-38]. The link between aP2 and metabolic and cardiovascular benefits in individuals carrying a rare haplo-insufficiency mutation in the aP2 locus, further validate the relevance of this pathway in humans [39,40]. These reports are therefore, consistent with our current findings showing reduced aP2 levels, improved glucose tolerance, and reduced adiposity in aging Pik3c3 mutants.

Our study demonstrates the role of autophagy in the regulation of the differentiated state and metabolic function of adipocytes that determine whole-body energy homeostasis. With the loss of autophagy function, reduced white adipose tissue differentiation, leading to markedly reduced gWAT in the aging mice. Additionally, gWAT acquired molecular signatures of brown adipose tissue with higher expression of *Ucp1* and *Pgc-1a* gene products in the middle and old-aged mice. Therefore, early block in the adipocyte differentiation program due to elevated Chop expression in young age might serve as a molecular driver for WAT browning with elevated levels of UCP1 and PGC-1a.

## METHODS

### Generation of Pik3c3 mutant mice

Vps34 (Pik3c3) gene knockout were generated on C57BL6 back ground through breeding of male FABp4-cre (B6.Cg-Tg(Fabp4-cre) 1Rev/J;Jackson Lab Stock # 005069) with the Pik3c3-floxed females (*Pik3c3*^tmic(EUCOMM)Wtsi/J^ ;Jackson Lab stock # 019081). The pups were genotyped at 21 days and the littermates are either Pik3c3^f/f^; Fabp4-Cre**^+^** or Pik3c3^f/f^; Fabp4-Cre- whereby Cre**^+^** were considered as mutants and Cre**^-^** were considered as control mice for subsequent analyses. Gonadal adipose tissues from the mice were analyzed at young (4 months), middle-aged (12 months), old-aged (22-24months). Mice were maintained in a pathogen-free environment at the Unit for Laboratory Animal Medicine (ULAM) facility at the University of Michigan (Ann Arbor, MI) until they were used for the study. All the experimental research in the current study has been approved by the University of Michigan University Committee on Use and Care of Animals (UCUCA).

### Isolation of adipose tissue

Careful inspection was done to exclude aged animals with cancer or lymphoma. Gonadal/epididymal fat pads were excised under sterile condition as previously described [29].

### Isolation of genomic DNA and genotyping

Genomic DNA (gDNA) was extracted from the tail biopsies from 3 weeks old pups using DNeasy Blood & Tissue Kit (Qiagene). Purified gDNA was used as a template for genotyping for LoxP and Cre genes using respective primers in PCR method. The PCR products were analysed in 1% agar electrophoresis. Similarly, adipocytes gDNA was isolated and analyzed for LoxP, Cre and Pik3c3 exon 4 deletions in PCR amplification method.

### RNA extraction and real-time quantitative PCR (RT-qPCR)

Adipose tissues were placed directly in RNA lysate buffer and RNA was extracted using the RNeasy kit (Qiagen). RNA was purified using RNeasy Lipid Tissue Midi Kit (Qiagen). Real-time PCR experiments were performed using QuantiTect SYBR green RT-PCR kit (Qiagen) using RNA samples with Corbett Rotor Gene 6000 Series (Qiagen, USA). Data analysis was performed by the comparative 2^ ^(-ddCT)^ method using Ct values.

### Western blotting

Protein quantities were analyzed by standard Western blotting technique. Briefly, 25-50 µg total proteins lysates were separated on Mini PROTEAN Precast Gels with 2 X Laemmli sample buffer (with 2.5% β-mercaptoethanol) to a final 30 µL volume. The proteins were transferred onto a PVDF-membrane and blocked with superblock solution and probed with anti-UCP1 (1:1000), anti-PGC1-a (1:1000), anti-aP2 (1:1000), and anti-Ppar-g (1:1000) from Cell Signaling Technology (Danvers, MA); anti-CHOP (1:500), anti-p62 (1:500) from Santa Cruz Biotechnology, and anti-Cebp-a (1:1000, Abcam) and anti-α Tubulin (1:5000, Abcam) for overnight at 4˚C. Anti-rabbit or anti-mouse horseradish peroxidase (HRP)-conjugated secondary antibodies (Santa Cruz Biotechnology) was used at a dilution of 1:5000 for 1 hr. at room temperature. The binding of specific antibodies was visualized via exposure to a photographic film after treating with enhanced chemiluminescence system reagents (Fisher Scientific, USA). The film was scanned and the band densities were quantified by ImageJ (NIH) software. The results were expressed as a relative ratio of the target protein to reference protein.

### Enzyme-Linked Immunosorbent Assay (ELISA)

Quantitation of Fet A in the serum, IL-6, and MCP-1 in the adipose tissue lysates were performed using respective ELISA kits (R&D Systems).

### Intraperitoneal Glucose Tolerance Test (IPGTT)

Mice were transferred to clean cages and fasted overnight for approximately 16 hrs. Mice were weighed to calculate and record the volume of 20% glucose solution required (2g of glucose/kg body mass) for IP injections for IPGTT: Volume of IP glucose injection (ml) =10 X body weight (g). Mice were restrained with an approved restrainer device with the tail exposed to score the tip of the tail with a sterilized scalpel blade. The first drop of blood was discarded and a small drop of blood is placed on the test strip of an animal blood glucose meter (Abbott Alpha TRAK 2 Blood Glucose Monitoring System) to record the fasting glucose level (t=0). Appropriate amounts of glucose were injected into the peritoneum as previously determined. The blood glucose levels were measured at 15, 30, 60 and 120 minutes following glucose injections and recorded. At the end of the experimental session, mice were placed in a clean cage with food and water.

### Homeostatic model assessment of Insulin Resistance (HOMA-IR)

HOMA-IR was calculated from fasting glucose and insulin concentrations with the standard formula: HOMA-IR= ((fasting glucose X fasting insulin)/22.5) [41]. Fasting insulin concentration in the serum was calculated by using ELISA kit (Crystal Chem. IL, USA).

### Statistical analyses

Results are expressed as Mean + SD in bar diagrams. The significance of difference between means with single variable was analyzed by Student’s t-test. The value of *p* <0.05 was considered to be statistically significant in all statistical analyses. All the statistical tests were performed with Graph Pad Prism 7 software Inc. (La Jolla, CA, USA)
